# Viable-but-Nonculturable Listeria monocytogenes and Salmonella enterica Serovar Thompson Induced by Chlorine Stress Remain Infectious

**DOI:** 10.1128/mBio.00540-18

**Published:** 2018-04-17

**Authors:** Callum J. Highmore, Jennifer C. Warner, Steve D. Rothwell, Sandra A. Wilks, C. William Keevil

**Affiliations:** a Centre for Biological Sciences, University of Southampton, Highfield Campus, Southampton, United Kingdom; b Vitacress Salads Ltd., Lower Link Farm, St Mary Bourne, Andover, United Kingdom; CEH-Oxford

**Keywords:** *Caenorhabditis elegans*, *Listeria*, *Salmonella*, VBNC, food-borne pathogens

## Abstract

The microbiological safety of fresh produce is monitored almost exclusively by culture-based detection methods. However, bacterial food-borne pathogens are known to enter a viable-but-nonculturable (VBNC) state in response to environmental stresses such as chlorine, which is commonly used for fresh produce decontamination. Here, complete VBNC induction of green fluorescent protein-tagged Listeria monocytogenes and Salmonella enterica serovar Thompson was achieved by exposure to 12 and 3 ppm chlorine, respectively. The pathogens were subjected to chlorine washing following incubation on spinach leaves. Culture data revealed that total viable L. monocytogenes and Salmonella Thompson populations became VBNC by 50 and 100 ppm chlorine, respectively, while enumeration by direct viable counting found that chlorine caused a <1-log reduction in viability. The pathogenicity of chlorine-induced VBNC L. monocytogenes and Salmonella Thompson was assessed by using Caenorhabditis elegans. Ingestion of VBNC pathogens by C. elegans resulted in a significant life span reduction (*P* = 0.0064 and *P* < 0.0001), and no significant difference between the life span reductions caused by the VBNC and culturable L. monocytogenes treatments was observed. L. monocytogenes was visualized beyond the nematode intestinal lumen, indicating resuscitation and cell invasion. These data emphasize the risk that VBNC food-borne pathogens could pose to public health should they continue to go undetected.

## IMPORTANCE

Many bacteria are known to enter a viable-but-nonculturable (VBNC) state in response to environmental stresses. VBNC cells cannot be detected by standard laboratory culture techniques, presenting a problem for the food industry, which uses these techniques to detect pathogen contaminants. This study found that chlorine, a sanitizer commonly used for fresh produce, induces a VBNC state in the food-borne pathogens Listeria monocytogenes and Salmonella enterica. It was also found that chlorine is ineffective at killing total populations of the pathogens. A life span reduction was observed in Caenorhabditis elegans that ingested these VBNC pathogens, with VBNC L. monocytogenes as infectious as its culturable counterpart. These data show that VBNC food-borne pathogens can both be generated and avoid detection by industrial practices while potentially retaining the ability to cause disease.

## INTRODUCTION

Entry into a viable-but-nonculturable (VBNC) state has been identified in a wide range of bacterial species and environmental stressors, including starvation, low temperature, antibiotic pressure, and oxidative stress (1–3). This survival state allows populations to persist and endure under harsher conditions than their culturable counterparts, including antibiotic tolerance and high temperatures (4). Despite the protection that the state provides for many bacterial pathogens, there are crucial gaps in the understanding of its underlying mechanisms and uncertainty regarding the infective potential of VBNC pathogens. This is particularly relevant to food-borne pathogens, where the industry relies almost exclusively on the use of culture recovery techniques to detect microbial contamination.

Food-borne disease presents a consistent but frequently preventable threat to public health and is responsible for an estimated 2.2 million deaths worldwide annually. In the United Kingdom, it is estimated that each year one million people suffer a food-borne illness, resulting in 500 deaths. In 2010, the bacterial food-borne pathogens Listeria monocytogenes and *Salmonella* spp. were responsible for more than half of these deaths following gastrointestinal infection (5). Another United Kingdom study spanning 17 years determined that in food-borne outbreaks, *Salmonella* spp. were responsible for the highest number of disease cases and the greatest proportion of deaths was caused by L. monocytogenes (6).

Fresh produce such as lettuce and spinach provides an effective vehicle for these pathogens, as they are often sold as ready-to-eat foods. As consumer habits are tending toward healthier eating with more fresh produce, the risk of disease outbreaks is increasing (7). In 2016, an outbreak of L. monocytogenes associated with packaged salads caused 19 cases, each resulting in hospitalization, across nine states in the United States (8). In the United Kingdom, an outbreak was caused by L. monocytogenes contaminating sandwiches sold at a hospital, affecting five pregnant women (9). Although *Salmonella* species outbreaks are proportionally less severe, they are farther reaching. One produce-associated outbreak of Salmonella enterica serovar Saintpaul resulted in 1,500 disease cases across 43 U.S. states, which hospitalized 21% of those affected and may have caused two deaths (10).

Despite their nonculturability, VBNC food-borne pathogens still pose a risk to consumers. While there is conflicting data on the pathogenicity of VBNC cells, there is evidence for their resuscitation under more favorable conditions, potentially allowing pathogens to cause disease prior to or even following ingestion by humans. Research carried out with L. monocytogenes has found that VBNC cells induced by starvation were avirulent when exposed to human adenocarcinoma cells but were resuscitated when inoculated into embryonated chicken eggs and regained virulence (11, 12). Similar results have been observed with S. enterica serovar Typhimurium, where VBNC cells induced by UV irradiation were unable to cause infection in a mouse model (13); however, another study using S. enterica serovar Oranienburg induced into the VBNC state by osmotic stress found that resuscitation could be achieved following injection into a mouse model (14). Other pathogens have been shown to retain aspects of their virulence while VBNC; the toxin genes of Shigella dysenteriae and Escherichia coli O157 have been detected while the bacteria are nonculturable (15, 16).

The parameters of the VBNC state and the infectivity of VBNC pathogens have been explored with a focus on VBNC induction via harsh conditions that bacteria are likely to encounter in a natural environment, but food production provides alternate stressors for food-borne pathogens. Chlorine is widely used to decontaminate fresh produce of both food-borne pathogens and spoilage bacteria. Previously, the efficacy of chlorine against L. monocytogenes has been measured by using culture techniques, reporting that there were no viable cells recovered after using 50 ppm chlorine (17). The presence of VBNC cells was not measured. Chlorine has been shown to induce the VBNC state in *Salmonella* Typhimurium biofilms (18). Further work concentrating on chlorinated drinking water and wastewater found that chlorine induces the VBNC state in a range of pathogens, including E. coli, *Salmonella* Typhimurium, and Helicobacter pylori (19, 20). The relevance of the VBNC state to food safety has recently been reviewed (21). However, it has yet to be shown whether chlorine-stressed pathogens remain infective in animals.

The mechanisms responsible for the antimicrobial activity of chlorine are not fully understood, though studies indicate that reactive chlorine species attack the bacterial inner membrane, where the dose of HOCl required for cell killing is similar to the dose required for ATP loss, loss of DNA replication, and prevention of protein transport across the inner membrane (22, 23).

This study simulated the passage of spinach contaminated with L. monocytogenes and S. enterica serovar Thompson from farm and processing to ingestion. In this way, VBNC induction of the pathogens by chlorine was assessed *in situ* on the spinach leaf phylloplane, comparing culture techniques to direct viable counts (with enumeration of both culturable and VBNC cells). The potential for infection by VBNC pathogens was then determined by using the animal model Caenorhabditis elegans.

## RESULTS

### Visualization of pathogen adherence to spinach phylloplane.

**FIG 1:**
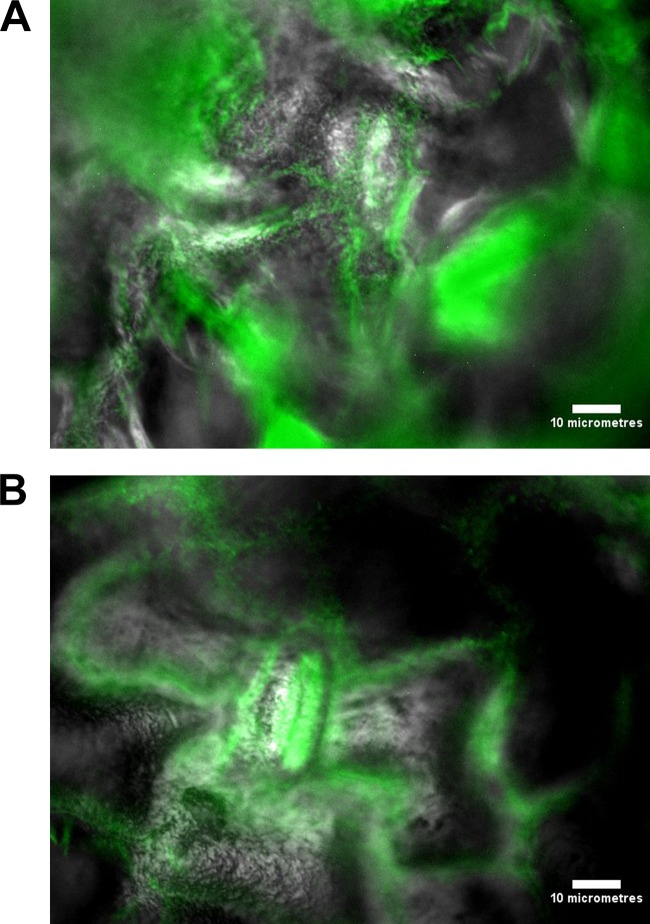
(A) Overlaid EDIC-EF micrographs of fluorescent L. monocytogenes adhering to the spinach phylloplane after 24 h of incubation. (B) Overlaid EDIC-EF micrographs of fluorescent Salmonella Thompson adhering to the spinach phylloplane after 24 h of incubation. Scale bars, 10 µm.

L. monocytogenes and Salmonella Thompson were visualized by episcopic differential interference contrast (EDIC)-epifluorescence (EF) microscopy following 24 h of incubation on the spinach phylloplane. Green fluorescence indicated that the pathogens were localized primarily inside the spinach stomata and at cell junctions. Compared with uninoculated control spinach leaves, both inoculated spinach samples possess a rough, uneven surface indicative of biofilm growth (Fig. 1).

### Induction of VBNC L. monocytogenes and Salmonella Thompson in chlorinated water.

**FIG 2:**
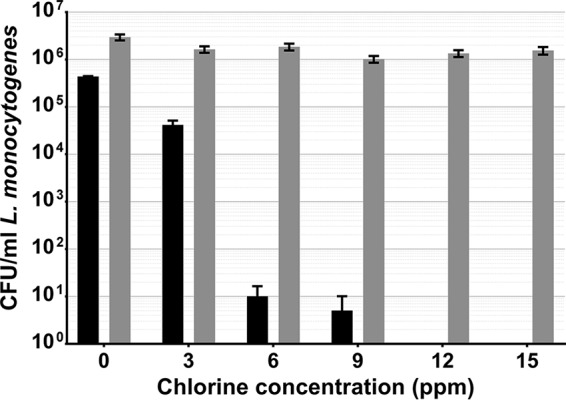
L. monocytogenes exposed to chlorinated water, cultured on selective media (black), and quantified by DVC (gray). Error bars indicate the SEM of two replicates.

L. monocytogenes became fully VBNC after 2 min of exposure to 12 ppm chlorine, with a just-under-1-log reduction of culturability at 3 ppm (*P* < 0.0001) and a >4-log reduction by 6 ppm (Fig. 2). Between 0 and 15 ppm, 47.64% of the viable cells counted by direct viable counting (DVC) were lost (*P* = 0.0075).

**FIG 3:**
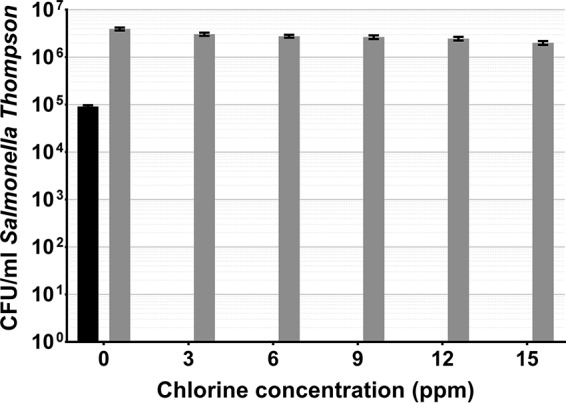
Salmonella Thompson exposed to chlorinated water, cultured on selective media (black), and quantified by DVC (gray). Error bars indicate the SEM of two replicates.

Salmonella Thompson became fully VBNC after 2 min of exposure to 3 ppm chlorine (*P* < 0.0001). Each increase in the chlorine concentration was met with a loss of Salmonella Thompson cells, with a 49% reduction between 0 and 15 ppm chlorine (*P* < 0.0001). There was also a 1.4-log difference between culturable cells and those enumerated by DVC (*P* < 0.0001) at 0 ppm chlorine (Fig. 3).

### Induction of VBNC L. monocytogenes and Salmonella Thompson adhering to the spinach phylloplane.

**FIG 4:**
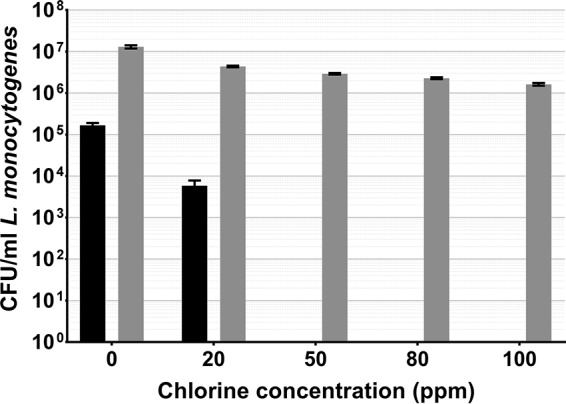
L. monocytogenes adhering to spinach leaves washed in chlorinated water, cultured on selective media (black), and quantified by DVC (gray). Error bars indicate the SEM of four replicates.

Spinach-adherent L. monocytogenes became fully VBNC after 2 min of exposure to 50 ppm chlorine, with a culturability reduction of 96.5% at 20 ppm. Direct viable counts declined with each increase in the chlorine concentration, where only the decrease between 20 and 50 ppm was not statistically significant. Despite this, there was <1-log reduction between 0 and 100 ppm. There was also a 1.7-log discrepancy between culture data and DVC data at 0 ppm (Fig. 4).

**FIG 5:**
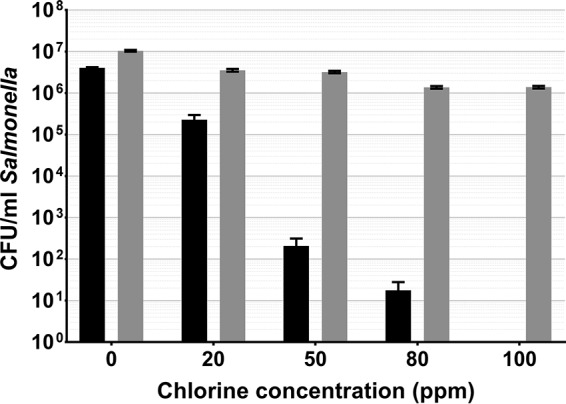
Salmonella Thompson adhering to spinach leaves washed in chlorinated water, cultured on selective media (black), and quantified by DVC (gray). Error bars indicate the SEM of four replicates.

Salmonella Thompson adhering to spinach leaves became fully VBNC after a 2-min exposure to 100 ppm chlorine, with a mean density of 207 CFU/ml at 50 ppm and 18 CFU/ml at 80 ppm (Fig. 5). Consistent with L. monocytogenes, a DVC reduction was observed with each increase in the chlorine concentration until a plateau was reached at 100 ppm. Again, there was a <1-log DVC reduction between 0 and 100 ppm (Fig. 5).

### Virulence of VBNC L. monocytogenes and Salmonella Thompson ingested by C. elegans*.*

**FIG 6:**
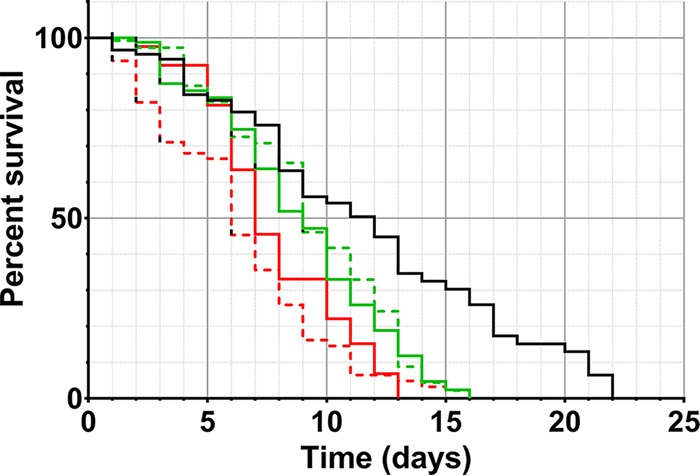
Survival of C. elegans exposed to culturable (solid line) and VBNC (broken line) L. monocytogenes (green) and Salmonella Thompson (red). E. coli Op50 (black) was used as a nonpathogenic control.

C. elegans that had only ingested E. coli Op50 survived for a maximum of 22 days. All of the worms exposed to culturable and VBNC L. monocytogenes died by day 16, with no statistically significant difference between the two conditions. C. elegans exposed to culturable Salmonella Thompson died by day 13, and worms exposed to VBNC Salmonella Thompson died by day 15. Significantly different nematode life span reductions were caused by E. coli Op50 and culturable L. monocytogenes (*P* = 0.0012) and by E. coli Op50 and VBNC L. monocytogenes (*P* = 0.0064), where the median life span of C. elegans feeding on E. coli Op50 was 12 days and only 9 days for both L. monocytogenes treatments. Similarly, ingestion of culturable (*P* < 0.0001) or VBNC (*P* < 0.0001) Salmonella Thompson significantly reduced the C. elegans life span compared with ingestion of the E. coli Op50 control. The median life spans of C. elegans worms that fed on culturable and VBNC Salmonella Thompson were 6 and 7 days, respectively, with a statistically significant difference observed between the two treatments (*P* = 0.0322) (Fig. 6).

**FIG 7:**
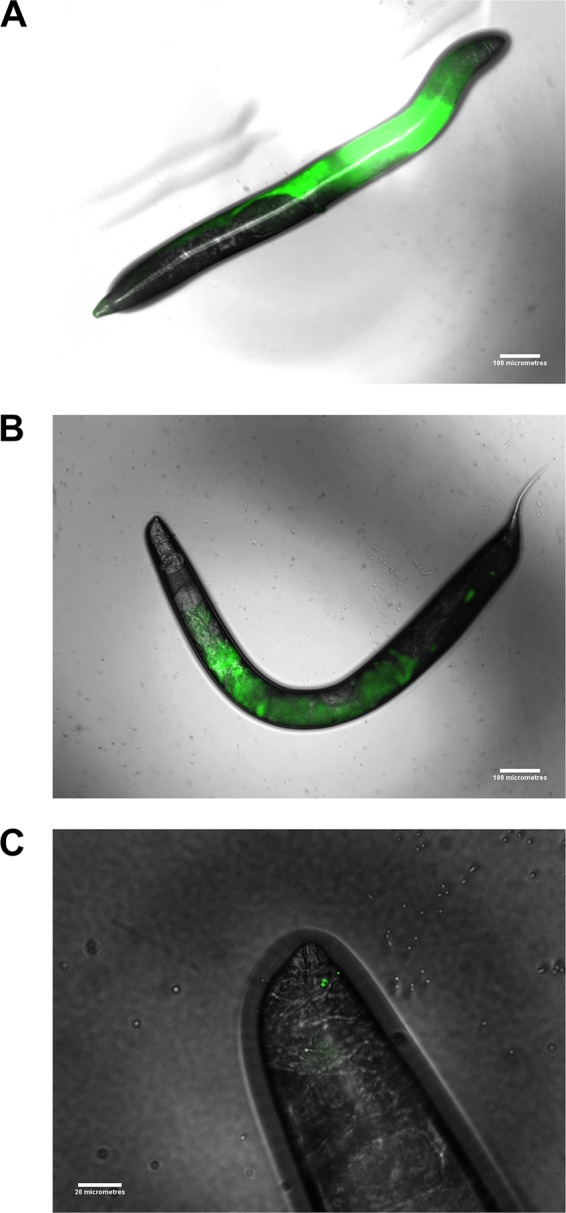
(A) Overlaid EDIC-EF micrographs of fluorescent VBNC L. monocytogenes ingested by C. elegans. (B) Overlaid EDIC-EF micrographs of fluorescent VBNC Salmonella Thompson ingested by C. elegans. Scale bars, 100 µm. (C) Overlaid EDIC-EF micrographs of fluorescent VBNC Salmonella Thompson ingested by C. elegans at the head of the nematode. Scale bar, 20 µm.

Green fluorescent protein (GFP) fluorescence from each pathogen assessed was observed filling the intestinal lumen of C. elegans (Fig. 7) and, in the case of L. monocytogenes, permeating the surrounding tissues (Fig. 7A). Pathogen cells were still visible when nematodes were returned to E. coli Op50 plates.

## DISCUSSION

As chlorine is commonly used in the agricultural industry to decontaminate fresh produce, food-borne pathogens will be exposed to the sanitizer during food production, both adhering to the phylloplane and detached in suspension. Here we show that in both cases, exposure to chlorine can induce the VBNC state in L. monocytogenes and Salmonella Thompson (Fig. 2 to 5). In water, L. monocytogenes becomes fully VBNC when exposed to 12 ppm chlorine, although 50 ppm is required following incubation on the spinach phylloplane (Fig. 2 and 4). Similarly, Salmonella Thompson becomes fully VBNC following exposure to 100 ppm chlorine on the phylloplane but only 3 ppm is required in chlorinated water (Fig. 3 and 5). This could largely be explained by the bacterial colonization of the phylloplane. Both pathogens are localized primarily in and around stomata and at cell junctions, thus potentially physically protected from the sanitizer.

A further benefit to phylloplane adherence is the facilitation of biofilm formation, where the production of an extracellular polysaccharide matrix presents a barrier to chlorine molecules. Previous studies have shown chlorine and hypochlorite to have limited penetrative ability in Pseudomonas aeruginosa and Klebsiella pneumoniae biofilms (24, 25), as well as in *Salmonella* biofilms (26). This effect could be supplemented by the autochthonous bacterial species present on the phylloplane. Nonfluorescent bacterial growth observed on the spinach cell surface indicates biofilm formation by indigenous species (Fig. 1), where an agonistic interaction with the inoculated food-borne pathogen may serve to reduce chlorine efficacy. These interactions could account for the relative decrease in sensitivity to chlorine observed in Salmonella Thompson on the phylloplane, where in double-distilled H_2_O (ddH_2_O) the pathogen lost culturability more easily than L. monocytogenes (Fig. 2 to 5). It was postulated in one study that when the food-borne pathogen E. coli O157 is attached to the spinach phylloplane, its biofilm-forming capability may be augmented by the presence of indigenous epiphytic bacteria (27). Despite the protective effect of biofilm, exposure to 5.5 ppm chlorine has previously been shown to induce the VBNC state in *Salmonella* biofilm (18).

This corroborates the findings of this study. The total population of L. monocytogenes and Salmonella Thompson lost culturability following exposure to 100 ppm chlorine (Fig. 4 and 5), where the approximately 1-log reduction in bacteria determined by DVC can be attributed to cell death by chlorine exposure. Here, that reduction resulted in 1.6 × 10^6^ CFU/ml VBNC L. monocytogenes and 1.4 × 10^6^ CFU/ml VBNC Salmonella Thompson. Typically in the agricultural industry, 90 ppm chlorine is used to wash fresh produce and is assumed to sanitize the food and the surrounding water. While these data show that an increase in the chlorine concentration does result in a loss of viable bacteria, the use of chlorine in industry is limited by the damage it causes to the food product, particularly leafy vegetables. Decontamination of food products by chlorination may be ubiquitous across food production; however, a wealth of research has shown chlorine to be ineffective at killing food-borne pathogens, including L. monocytogenes and E. coli O157 inoculated onto lettuce (28, 29).

The initial bacterial inoculum concentrations reflect both previous research assessing contamination of crop plants by food-borne pathogens (30, 31) and the level of contamination previously detected in vegetables affected by bacterial soft rot collected from a marketplace in the United States (32). From contaminated spinach, 3 × 10^5^ suspected *Salmonella* colonies/ml of wash water were detected, and using enrichment broth, 1.7 × 10^7^ and 8.6 × 10^8^ CFU/ml were detected in healthy and rotting spinach, respectively. In this study, biofilms were grown on the spinach phylloplane for 24 h at room temperature, so the resulting bacterial population is indicative of the level of contamination that would be seen in the field.

In water, the relatively greater sensitivity to chlorine observed in Salmonella Thompson (Fig. 2 and 3) could be due to the nature of the damage caused by reactive chlorine species in bacteria. Chlorine is thought to cause bacterial cell death by impeding the functions of the inner membrane (22). Salmonella Thompson is Gram-negative, whereas L. monocytogenes is Gram-positive and the Gram-positive thick peptidoglycan layer could influence susceptibility to chlorine stress. Previously, it has been shown that inactivation by exposure to singlet oxygen is affected by the presence of the peptidoglycan layer (33).

The data obtained show a pronounced difference between untreated cells quantified by culture and by DVC, particularly in Fig. 4. In this case, it could be that the osmotic stress placed upon L. monocytogenes in ddH_2_O resulted in some loss of culturability without exposure to chlorine. It is also possible that the discrepancy is a consequence of the assumption that cells are evenly distributed across each microscope slide.

The data obtained in this study suggest that the chlorine-mediated killing of bacteria observed in previous research can be attributed, in part, to VBNC induction by chlorine. In the food industry, the use of chlorine to decontaminate minimally processed food results in the inability of “gold standard” culture techniques to detect food-borne pathogens, which may then go on to cause disease outbreaks. As similar work has not yet been carried out with alternative methods of fresh produce decontamination, their efficacies may also be reduced by VBNC induction. Studies assessing the efficacy of sanitizers such as ozone (34, 35), gamma (36) or UV (37) irradiation, and ultrasound (38–40) routinely use culture-based bacterial enumeration exclusively, so the contribution of VBNC bacteria has not been explored. However, previous studies have observed that these exposures to UV irradiation and ultrasound can also result in VBNC induction in different pathogens (41, 42). In finding alternative decontamination treatments, industry is further restricted as it must effectively kill bacteria without inducing the VBNC state and without compromising the quality of the food product.

The nematode killing assay revealed that there is no difference in the virulence of L. monocytogenes in the culturable and VBNC states and that both cause a reduction in the C. elegans life span (Fig. 6). Previous work with L. monocytogenes has provided evidence that the pathogen is avirulent in the VBNC state (11). The results of this study could contradict this for several reasons; this study focused on VBNC induction by chlorine exposure, whereas Cappelier et al. (11) generated VBNC cells via starvation. Using human cell lines as a model, virulence was previously measured by assessing the invasive properties of L. monocytogenes and it was injected into the bloodstream in a mouse model. In this study, infection was modeled in C. elegans by ingestion and infection of the gastrointestinal tract. It has been shown that VBNC E. coli O157 maintains the expression of its Shiga-like toxin genes when it is VBNC (15), so while there is limited research on L. monocytogenes, it is possible that toxin expression causes disease in the digestive tract while cell invasion in the VBNC state is impaired.

The suggestion that there are differences in the VBNC states of the same pathogen dependent on the method of VBNC induction has not been explored but could present further challenges for the food industry. Prior to harvest, the phylloplane is a harsh environment for bacteria, with exposure to UV radiation and limited moisture providing conditions that could induce the VBNC survival state in food-borne pathogens before exposure to chlorination. There is evidence of this, as VBNC induction has been shown to occur in E. coli O157 on the lettuce phylloplane in response to low temperatures (2). While these data show that VBNC L. monocytogenes induced by chlorine can cause disease, VBNC pathogens induced by physical stimuli on the phylloplane may require a separate assessment comparing VBNC expression profiles, where the fundamental mechanisms of the state have yet to be fully understood.

Corroborating previous studies (43), C. elegans feeding on Salmonella Thompson was also found to significantly reduce the worm’s life span, where worms fed on culturable Salmonella Thompson died within 13 days and those fed on VBNC Salmonella Thompson died within 15 days (Fig. 6). By comparing them to one another, it was determined that a significantly greater reduction in the C. elegans life span is achieved by using culturable Salmonella Thompson (*P* = 0.0322). This indicates that while the pathogen is still virulent in the animal model, it does lose some infectivity in the VBNC state. Research on the cell invasion ability of VBNC *Salmonella* Typhimurium has indicated that VBNC cells have an impaired ability to invade epithelia (44) and those induced by antibiotic pressure are unable to cause disease in mice (45). Conversely, immunocompromised mice that ingested VBNC *Salmonella* Oranienburg were affected by the pathogen, suggesting that there is still a risk of infection by VBNC *Salmonella* under certain conditions (14). The relative success of VBNC L. monocytogenes in reducing the C. elegans life span to a degree similar to that of its culturable counterpart could be due to the ability of the pathogen to grow at lower temperatures (46). VBNC Salmonella Thompson may require a higher temperature, such as the mammalian core temperature of 37°C, to more effectively resuscitate and establish infection.

Both pathogens in the VBNC state could be seen fluorescing inside the intestinal lumen of C. elegans (Fig. 7). L. monocytogenes completely fills the intestinal tract and has invaded the surrounding tissues, with the ovary of the nematode masking the terminal end of the tract (Fig. 7A). The high level of fluorescence observed, even when nematodes are removed from the pathogen food source, provides evidence that the bacteria have colonized the gut, which may suggest resuscitation once inside a host. This is supported by the fluorescence extending beyond the intestine, which is consistent with the cell invasion that occurs upon L. monocytogenes infection (47). A similar phenomenon has been observed in L. monocytogenes, where resuscitation occurred following introduction into embryonated eggs but not following introduction into nonembryonated eggs (12).

The differences observed between C. elegans infections by S. enterica and L. monocytogenes have also been observed in *Tetrahymena* (48). Salmonella Thompson was released in vesicles from the protozoan, while L. monocytogenes was digested. In this case, the authors observed that ingestion by *Tetrahymena* protects Salmonella Thompson from environmental stresses. In this study, Salmonella Thompson accumulated in the intestine at the pharyngeal-intestinal valve (Fig. 7B), resembling *Salmonella* infection in vertebrate hosts, where attachment to the apical surface of epithelial cells takes place (49). The different interactions of both food-borne pathogens with the C. elegans host may indicate that resuscitation has also taken place in VBNC Salmonella Thompson, resulting in its virulence in the nematode. These data support the use of the C. elegans invertebrate model for the study of VBNC food-borne pathogens; it is more cost and space efficient than the use of vertebrate models and is free from ethical constrains. In addition, the presence of a well-defined nervous system and digestive tract, with a mouth, a pharynx that pumps the food into the intestines, a digestive system that enables the worm to process the food, and an excretory system, makes this animal model more applicable to higher organisms than others such as the unicellular amoebal and wax moth larva infectivity models.

Preliminary work conducted in this study is consistent with resuscitation of VBNC pathogens inside the host; when assessed using a nematode killing assay, GFP-tagged Salmonella Thompson strain RM2311 was not found to reduce the C. elegans life span. However, C. elegans worms that fed on Salmonella Thompson died rapidly from day 12, which could be a result of colonization or, in the case of VBNC cells, resuscitation (data not shown). Conversely, Salmonella Thompson strain NCTC 2252 was shown to reduce the C. elegans life span (Fig. 6), where the difference in infectivity may be a result of the fitness cost of GFP expression by the pathogen (50).

The data obtained in this study do not discern whether VBNC L. monocytogenes and Salmonella Thompson cause disease by resuscitation stimulated by ingestion by a host or by continued expression of virulence factors while in the VBNC state. However, they do provide evidence that the use of chlorine to decontaminate fresh produce is not only ineffective but permits virulent food-borne pathogens to reach the public undetected by standard methods. Outbreaks of food-borne disease where no food vehicle can be identified do occur (51), and it is possible that the VBNC state plays an important role. Consequently, new methods are required to rapidly detect VBNC pathogens, which are still capable of causing disease despite accepted sanitization procedures, to protect public health. Indeed, it may be better not to sanitize foodstuffs and rely instead on rapid pathogen detection methods and positive release of those foodstuffs deemed safe for human consumption.

## MATERIALS AND METHODS

### Bacterial strains.

The bacteria used in this study were L. monocytogenes Scott A expressing GFP on plasmid pPL3-GFP and S. enterica serovar Thompson strains NCTC 2252 and RM2311. Salmonella Thompson RM2311 expresses GFP on plasmid pWM1007, which also contains a kanamycin resistance gene (52, 53). Both were cultured for 18 h at 37°C in brain heart infusion broth (BHIB; Oxoid, United Kingdom). L. monocytogenes was cultured on agar by using the selective medium PALCAM (Oxoid, United Kingdom) with *Listeria* selective supplement (Sigma-Aldrich, United States), and S. enterica was cultured on agar by using CHROMagar Salmonella Plus with its cognate supplement (CHROMagar, France). E. coli Op50 was used as a nonpathogenic control in the nematode killing assay. It was cultured in Luria-Bertani broth (Formedium, United Kingdom) for 18 h at 37°C prior to use.

### Leaf samples.

The leaf samples used were raw, unwashed spinach leaves supplied by Vitacress Salads Ltd., United Kingdom. Leaves were inoculated within 48 h of delivery. Twenty-five-gram leaf samples were placed in a Stomacher bag (Interscience, France) and inoculated with 1 ml of bacteria at a concentration of 5 × 10^7^ CFU/ml of BHIB. Inoculated samples were shaken vigorously and incubated at 22°C for 24 h prior to being washed with chlorine.

### Chlorinated washing water samples.

A stock solution of 2,500 ppm free chlorine was produced by dissolving one Haz-Tab (Guest Medical, United Kingdom) in 1 liter of ddH_2_O, which was further diluted in ddH_2_O to generate working solutions. Bacterial suspensions of 10^8^ CFU in phosphate-buffered saline (PBS; Oxoid, United Kingdom) were inoculated into 50 ml of ddH_2_O in a Stomacher bag to which 50 ml of the appropriate chlorine dilution was added. The sample was shaken vigorously for 2 min and then filtered through a 0.22-µm-pore-size mixed cellulose ester membrane (Millipore, USA) by vacuum filtration. Bacteria were removed from the membrane by placement in another Stomacher bag with 100 ml of PBS and shaken with a Pulsifier (Microgen, United Kingdom) for 30 s, producing a final concentration of 10^6^ CFU/ml. Samples were then taken for culture and DVC.

### Spinach.

Following 24 h of incubation, 225 ml of ddH_2_O containing the appropriate volume of chlorine solution was added to inoculated spinach samples. Samples were vigorously shaken for 2 min, and the liquid was discarded, retaining the leaf samples; 225 ml of PBS was then added, and the bag was shaken in the Pulsifier for 30 s. Samples of the resulting bacterial suspension were then taken for culture and DVC.

### DVC and visualization of samples.

Samples taken for DVC were concentrated by centrifuging a 10-ml sample for 15 min at 4,000 rpm with a Heraeus Megafuge 1.0. The sample was then resuspended in 1 ml of PBS. To aid visualization, samples were subjected to cell elongation by a modification of the method of Juhna et al. (54). The 1-ml sample was added to 4 ml of ddH_2_O, 5 ml of R2 broth (0.1% [wt/vol] peptone, 0.05% [wt/vol] yeast extract, 0.05% [wt/vol] glucose, 0.05% [wt/vol] starch, 0.03% [wt/vol] potassium dihydrogen phosphate, 0.03% [wt/vol] sodium pyruvate, 0.0024% [wt/vol] magnesium sulfate), and 10 µl of pipemidic acid at a concentration of 10 µg/ml. The suspension was incubated for 18 h at 22°C in darkness. The suspension was concentrated prior to DVC in the same manner as before.

All samples were imaged by using EDIC-EF microscopy (55) and a QImaging Retiga EXi camera. Bacteria were quantified by counting visible cells across at least 30 fields of view per sample. Images were merged with ImageJ.

### C. elegans killing assay.

C. elegans worms were maintained on 5-cm nematode growth medium (NGM) agar plates prepared in accordance with standard methods (56) with a lawn of E. coli Op50. To prepare an experimental plate, 50 µl of E. coli Op50, L. monocytogenes, or Salmonella Thompson culture was added to the center of the plate and it was incubated at 22°C for 24 h. To produce VBNC cells, cultures of L. monocytogenes and Salmonella Thompson were pelleted by centrifugation and resuspended in 10 ml of a 200 ppm chlorine solution for 30 min. Chlorinated water was removed by vacuum filtration as described above, and bacteria were removed from the membrane by vortexing in 1 ml of PBS for 2 min (57), concentrating the sample to compensate for the growth of the culturable counterparts on the NGM plate. Plates were then inoculated with 50 µl of VBNC cells and incubated at 22°C for 24 h. VBNC cells were plated on selective media to verify the VBNC state.

C. elegans worms were transferred to experimental plates at the L4 stage. Twenty worms were used per plate, and each condition was tested with at least four replicates. Nematodes were counted daily and transferred to fresh plates every other day. Nematodes that did not respond when prodded with a pick were considered dead.

### Statistical analyses.

Culture data and DVC were separately subjected to one-way analysis of variance with Tukey’s multiple-comparison test. Comparisons of culture and DVC data were done with multiple *t* tests. Nematode killing assay data were analyzed by using the survival curve comparison Mantel-Cox test. All statistical analyses were done with GraphPad Prism 7.
